# Physiological Associations between Vitamin B Deficiency and Diabetic Kidney Disease

**DOI:** 10.3390/biomedicines11041153

**Published:** 2023-04-11

**Authors:** Henry H. L. Wu, Thomas McDonnell, Rajkumar Chinnadurai

**Affiliations:** 1 Renal Research Laboratory, Kolling Institute of Medical Research, Royal North Shore Hospital, The University of Sydney, Sydney, NSW 2065, Australia; 2 Department of Renal Medicine, Northern Care Alliance NHS Foundation Trust, Salford M6 8HD, UK; thomas.mcdonnell@nca.nhs.uk (T.M.);; 3 Faculty of Biology, Medicine & Health, The University of Manchester, Manchester M1 7HR, UK

**Keywords:** vitamin B, vitamin B deficiency, diabetes mellitus, chronic kidney disease, diabetic kidney disease

## Abstract

The number of people living with chronic kidney disease (CKD) is growing as our global population continues to expand. With aging, diabetes, and cardiovascular disease being major harbingers of kidney disease, the number of people diagnosed with diabetic kidney disease (DKD) has grown concurrently. Poor clinical outcomes in DKD could be influenced by an array of factors—inadequate glycemic control, obesity, metabolic acidosis, anemia, cellular senescence, infection and inflammation, cognitive impairment, reduced physical exercise threshold, and, importantly, malnutrition contributing to protein-energy wasting, sarcopenia, and frailty. Amongst the various causes of malnutrition in DKD, the metabolic mechanisms of vitamin B (B1 (Thiamine), B2 (Riboflavin), B3 (Niacin/Nicotinamide), B5 (Pantothenic Acid), B6 (Pyridoxine), B8 (Biotin), B9 (Folate), and B12 (Cobalamin)) deficiency and its clinical impact has garnered greater scientific interest over the past decade. There remains extensive debate on the biochemical intricacies of vitamin B metabolic pathways and how their deficiencies may affect the development of CKD, diabetes, and subsequently DKD, and vice-versa. Our article provides a review of updated evidence on the biochemical and physiological properties of the vitamin B sub-forms in normal states, and how vitamin B deficiency and defects in their metabolic pathways may influence CKD/DKD pathophysiology, and in reverse how CKD/DKD progression may affect vitamin B metabolism. We hope our article increases awareness of vitamin B deficiency in DKD and the complex physiological associations that exist between vitamin B deficiency, diabetes, and CKD. Further research efforts are needed going forward to address the knowledge gaps on this topic.

## 1. Introduction

Chronic kidney disease (CKD), defined by the presence of reduced estimated glomerular filtration rate (eGFR) and/or increased urinary albumin excretion for 3 months or more, is commonly observed amongst people with type 1 (T1DM) or type 2 diabetes mellitus (T2DM) [1,2,3]. Diabetic kidney disease (DKD) is considered as one of the key causes of CKD [1,2,3,4]. The majority of patients diagnosed with DKD are from an older age cohort, living with frailty and other co-morbidities in addition to diabetes and kidney disease [1,2,4]. Many will have progressive loss of kidney function and eventually develop kidney failure. In fact, DKD is currently the primary cause of kidney failure across the globe [5,6]. Recent United States Renal Data System annual reports have highlighted diabetes as the primary etiology in nearly half of all patients diagnosed with kidney failure in the United States [7,8]. Though the strongest risk factor for DKD progression is albuminuria, other factors such as poor glycemic control, obesity, metabolic acidosis, anemia, cardiovascular disease, cellular senescence, infection and inflammation, cognitive impairment, reduced physical exercise threshold, and malnutrition are also considered important factors for progression towards kidney failure in the DKD population [3,9,10,11,12,13,14,15]. 

Many CKD patients live with micronutrient deficiencies, with vitamin B being a major micronutrient of note in terms of vitamin B deficiency. The B-class vitamins are a class of water-soluble vitamins that play important roles in cellular metabolism and homeostasis, antioxidation, as well as the synthesis of red blood cells [16,17]. Vitamin B is predominantly sourced from dietary intake [17]. Since they are water-soluble, they are generally poorly stored within the human body. Excess vitamin B would be readily excreted from urine, although individual absorption, use, and metabolism may vary between individuals [16,17,18]. Vitamin B deficiency, amongst other factors, may contribute towards the development of CKD, considering the progressing inflammatory and oxidative stress processes that are associated with impairment of kidney function and development of kidney histopathology. An emerging topic of discussion is the association between each vitamin B sub-form (B1 (Thiamine), B2 (Riboflavin), B3 (Niacin/Nicotinamide), B5 (Pantothenic Acid), B6 (Pyridoxine), B8 (Biotin), B9 (Folate), and B12 (Cobalamin)) and CKD. With the additional presence of diabetes, in addition to CKD and other co-morbidities, there could be more issues with absorption (i.e., gastroparesis being a complication of diabetes) of some vitamin B sub-forms alongside increased demands for energy production [18]. Vitamin B deficiency is likely inevitable without adequate supplementation in place. A meticulous approach towards vitamin B supplementation may be indicated, due to the body’s worsened ability to tolerate effects from over-supplementation and achieve cellular homeostasis during diseased states [19,20]. 

There is an increasing amount of evidence pointing out significant physiological links between vitamin B metabolism, vitamin B deficiency, and CKD. For some vitamin B sub-forms, numerous studies have extended their investigation of these associations within the DKD context in recent times—where importantly the impact of diabetes is added into this discussion. We provide an updated review aiming to evaluate the biochemical and physiological properties of each vitamin B sub-form in normal states and how their functions and metabolism may affect and/or could be affected by the development of DKD. We hope our article increases awareness of vitamin B deficiency in DKD and the complex physiological associations that exist between vitamin B deficiency, diabetes, and CKD. Given emerging evidence and remaining uncertainties with a relative paucity of observational data within this specific topic, further research efforts are needed going forward to address knowledge gaps.

The study selection for this narrative review encompassed the input of the following search terms: “Vitamin B”, “Vitamin B1”, “Vitamin B2”, “Vitamin B3”, “Vitamin B5”, “Vitamin B6”, “Vitamin B8”, “Vitamin B9”, “Vitamin B12”, “Thiamine”, “Riboflavin”, “Niacin”, “Pantothenic Acid”, “Pyridoxine”, “Biotin”, “Folate”, “Cobalamin”, “Deficiency”, “Diabetes”, “Diabetes Mellitus”, “Chronic Kidney Disease”, “Diabetic Kidney Disease”, and “Diabetic Nephropathy” into search engines, including PubMed, Web of Science, EMBASE, Google Scholar, and Medline-ProQuest. The search and selection process for the references that are included in this article were independently performed by the three authors of this review (H.H.L.W., T.M., R.C.). 

## 2. Vitamin B1 (Thiamine)

**Figure 1:**
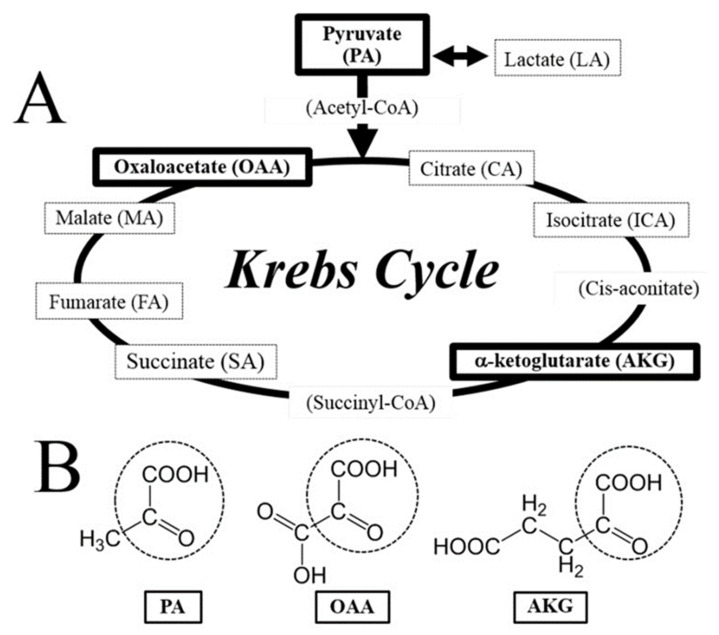
The citric acid (Krebs) cycle. Figure obtained from [25]. (**A**) Compounds involved in the Krebs cycle and (**B**) common chemical structure of neuroprotective krebs cycle intermediate [pyruvate (PA), oxaloacetate (OAA), and α-ketoglutarate (AKG)]. Note that PA, OAA, and AKG have the common chemical structure of the α-keto acid group—indicated by the dotted circle—with which molecules can directly react with hydrogen peroxide.

The condensation process of pyrimidine and thiazole rings forms the vitamin B1 compound, otherwise known as thiamine. The key functional forms of thiamine are thiamine pyrophosphate (TPP), monophosphate, and triphosphate (TTP), which are formed when thiamine combines with phosphate to form esters. High temperatures, high pH, oxidants, and ultraviolet radiation will destroy the thiamine structure, given its labile nature [21]. Thiamine is abundant in lean pork, yeast, and legumes. When these foods are cooked with water, significant amounts of thiamine from these foods would be released, as it is water-soluble [21]. In the human body, the small intestine is the organ where thiamine can be absorbed by active and passive processes. Thiamine in plasma is mainly albumin-bound, and the vast majority appears as TPP [22]. TPP has a key role in energy metabolism and adenosine triphosphate (ATP) production [21,22]. A large number of chemical reactions involving carbohydrate metabolism occurs with TPP as co-enzyme, which includes the oxidative decarboxylation of α-ketoacids and decarboxylation of alpha-ketoglutarate to succinyl-coenzyme A (CoA) in the citric acid (Krebs) cycle (Figure 1) [23,24,25]. This process is important for gamma-aminobutyric acid (GABA) production, as GABA is an inhibiting neurotransmitter in the brain [21,22,23,24,25]. A co-enzyme for pyruvate dehydrogenase, TPP also catalyzes the conversion of pyruvate to acetyl-CoA [21,22,23,24,25]. When thiamine deficiency is present, there is insufficient pyruvate conversion to acetyl-CoA through the citric acid cycle resulting in pyruvate accumulation. Pyruvate is shunted down the anaerobic pathway with increased generation of lactate and significantly less ATP production [21,22,23,25]. Thiamine plays a significant role in lactate utilization, and the presence of lactic acidosis can be thiamine-responsive [23]. Furthermore, thiamine is a co-enzyme for the transketolase reaction in the pentose phosphate pathway [24,25]. In myelinated structures of nervous tissues, levels of transketolase are high, which may explain the presence of peripheral neuropathy with thiamine deficiency, as seen in “dry” beriberi syndrome [26]. 

Thiamine availability and activity levels may be inhibited by folate deficiency, thiaminases, protein-energy malnutrition, and alcohol [21]. Numerous medications may affect thiamine metabolism—aminoglycosides, cephalosporins, fluoroquinolones, loop diuretics, penicillins, phenytoin, sulfonamide derivatives, tetracycline derivatives, and trimethorprim [21]. Thiamine metabolism and action may also be affected by inborn errors of thiamine transporters, which have become recognized to a greater extent over recent years [27]. Diabetes in SLC19A2 defects, peripheral neuropathy and encephalopathy in SLC19A3, SLC25A19, and TPK1 genetic defects, megaloblastic anemia, and deafness are the five phenotypes associated with inborn errors of thiamine transporters [27]. When thiamine is catabolized, many metabolites produced are urinary excreted [21]. 

There are recent advances in our basic understanding of the effects of thiamine deficiency on DKD and vice-versa. Thiamine, TPP, and TMP transporters may have an abnormal expression in diabetes [28,29,30]. Increased levels of the thiamine transport protein, isoform 1 (THTR1), which is encoded by SLC19A2, are likely to be present in red blood cells (RBCs) and mononuclear leukocytes of patients with diabetes in comparison to healthy subjects [28,29,30,31]. This occurs as the result of a homeostatic response from RBC precursors, reticulocytes, and erythroblasts, a thiamine-deficient environment sensitively noted by leukocytes in a diabetic state [29,32]. What is interesting is that there are different responses of THTR1 expression across the various cell types in diabetes [29,32]. Whereas RBC precursors and leukocytes appear to upregulate THTR1 expression in response to decreased thiamine availability, epithelial cells in the kidney tubules conversely are likely to have decreased expression of THTR1 in a hyperglycemic environment [33,34,35]. Basic studies highlighted decreased THTR1 levels in in vitro cultured human tubular epithelial HK-2 cells with high glucose concentration [36,37]. Amongst streptozotocin-induced diabetic rats, an eight-fold increase in renal clearance of thiamine associated with decreased tubular reuptake of thiamine was observed [36,37]. This was confirmed in human samples, which identified decreased levels of thiamine plasma concentration in patients with T1DM and T2DM [31,38]. Urinary thiamine excretion preceded microalbuminuria [33,34,35,39]. Compared to individuals with stable kidney function, human studies identified that patients with microalbuminuria and a decline in eGFR had a higher fractional excretion of thiamine [40]. An increased accumulation of soluble vascular cell adhesion molecule-1 and von Willebrand factor, markers of vascular inflammation and endothelial cell damage, is caused by thiamine deficiency [29,41]. These inflammatory cytokines and growth factors are a key part of contributing towards the loss of glomerular endothelial glycocalyx in DKD leading to the leakage of albumin (as well as thiamine) into glomerular filtrate at a rate that overwhelms the re-absorptive and metabolic capacity of the proximal tubular epithelium, as reflected by the presence of persistent microalbuminuria [31,33,42]. Given the indirect roles of transketolase in antioxidant defense within the oxidative and reductive pentosephosphate pathways, abnormal expression of transketolase with thiamine deficiency weakens enzymatic defense against glycation and its ability to counter metabolic stress, resulting in further acceleration of vascular dysfunction and progression of DKD [43,44,45,46].

## 3. Vitamin B2 (Riboflavin) 

Vitamin B2 (riboflavin) is an alloxazine derivative in which flavin mononucleotide (FMN) and flavin adenine dinucleotide (FAD) are its main active compounds. FMN is an alloxazine ring combined with ribitol and phosphate whilst the FMN molecule with the addition of an activated adenosine monophosphate forms FAD [47]. Compared to other vitamin B sub-forms, riboflavin is modestly soluble in water, appears more structurally stable in hotter temperatures, and is photosensitive [47]. Many plant and animal food products contain riboflavin, including bread, broccoli, cereals, eggs, milk, and lean meats [48]. Absorption occurs in the proximal jejunum, through an active sodium and glucose-dependent absorption pathway that is vital for the transport of small quantities of riboflavin, whilst the rest are absorbed across the intestine via passive diffusion facilitated by bile salts [47,48]. Following cellular uptake, flavokinase and FAD synthetase would transform riboflavin to FMN and the more abundant FAD, acting as cofactors to flavoproteins [47,48]. Riboflavin metabolites are excreted in urine. Flavoenzymes are involved in numerous oxidation-reduction reactions that are necessary for the intermediary metabolism of carbohydrates, amino acids, and lipids in addition to supporting cellular antioxidant potential [49,50]. 

Numerous medications are well-documented to interfere with riboflavin activity—anticholinergic drugs, tetracyclines, and probenecid may inhibit intestinal absorption of riboflavin; doxorubicin deactivates riboflavin and depletes its levels; tricyclic antidepressants, phenothiazines, phenytoin, and methotrexate can inhibit riboflavin metabolic actions; thiazide diuretics increase riboflavin urinary excretion (probenecid decreases urinary excretion) [47]. 

Kidney damage has been considered as one of the major abnormalities amongst growth retardation, anemia, skin lesions, and neurodegenerative changes when riboflavin deficiency is observed, as a result of dietary deprivation or periods of pathological or physiological stress [51,52]. Whilst the links between riboflavin and its protective action against oxidative stress are well-examined, there remains a paucity of evidence discussing oxidative stress and its role in the progression of diabetes and DKD with riboflavin deficiency. Nevertheless, a mice study by Alam and colleagues [53] noted riboflavin ameliorates oxidative stress and DNA damage in diabetic mice, where increased riboflavin levels following supplementation improved urea, creatinine, and antioxidant status of their kidneys. Significantly, greater recovery of glutathione reductase, an antioxidant enzyme catalyzing the reduction of glutathione disulfide to the sulfhydryl form glutathione, was observed. This is a critical molecule in resisting oxidative stress and maintaining the reducing environment of kidney cells [54,55]. Reduction in lipid peroxidation, protein oxidation, GST, and sulfhydryl levels in the kidneys were observed following riboflavin supplementation. The study by Alam and colleagues [53] also highlighted an improved histopathological outlook in the kidneys of mice with diabetes following riboflavin supplementation, in which mice with more severe diabetes exhibited less diabetes-induced structural aberrations attributed to the effects of riboflavin. The histopathological analysis also revealed riboflavin dose-dependent cellular DNA damage recovery. Further work is anticipated to explore and validate the pathophysiological associations between riboflavin, diabetes, and DKD, in particular the impact of riboflavin deficiency on oxidative stress build-up within this setting. 

## 4. Vitamin B3 (Niacin) 

Vitamin B3 (niacin) is a generic term for nicotinic acid and nicotinamide. Pyridine nucleotides, nicotinamide adenine dinucleotide (NAD), and NAD phosphate (NADP) are active co-enzymes of niacin [56,57]. The main forms of niacin are obtained from food sources with high levels of NAD and NADP, including coffee, fish, legumes, meat, and tea [56]. A precursor of niacin, tryptophan intake alone through food sources may be enough by itself to fulfill the requirements to avoid niacin deficiency [56,58]. Tryptophan is hydrolyzed in the intestine to form nicotinamide and then nicotinic acid [56,57,58]. However, niacin binding to carbohydrate and peptide macromolecules would reduce its bioavailability [59]. Passive diffusion is the most efficient mechanism of intestinal absorption, even for large amounts of niacin [56,57,58,59]. 

Following absorption, the liver and erythrocytes rapidly remove niacin from plasma, converting it to coenzyme forms [56,57]. Niacin storage in tissues is limited [56]. Given most of the absorbed niacin is stored in liver, excess niacin is methylated in the liver and the methylated metabolites are urinary excreted [57]. Pyridine nucleotides are involved in many enzymatic reactions (at least 200)—including NAD that is mainly involved in catabolic reactions such as oxidation of energy substrates and NADP that is primarily associated with biosynthetic processes such as those for steroids [60,61]. Coenzymes such as NAD and NADP are important elements for the metabolism of amino acid, carbohydrates, and fatty acids [62]. Niacin has a close metabolic association with other vitamin B sub-forms, namely vitamin B6 (pyridoxine) and riboflavin given both are instrumental for niacin synthesis from tryptophan [63]. In reverse, niacin is necessary for the synthesis of vitamin B6 (pyridoxine) and riboflavin, as well as vitamin B9 (folate) [56,57,63]. Synthesized niacin can reduce serum total cholesterol, increase the high-density lipoprotein (HDL)-cholesterol fraction, and decrease serum low-density lipoprotein (LDL) and very low-density lipoprotein (VLDL) fractions and triglycerides [64]. Early data have demonstrated improvements in lipidemia with niacin supplementation in T2DM patients, though niacin supplementation has not shown effects on improving glycemic levels [65,66]. The mechanisms of how niacin impacts lipid and glucose metabolism are complex and remain not fully clear, though it is shown that this differs from one fraction to another [67,68]. Further validation of these relationships is required. Statins, which increase the risk of myopathy and rhabdomyolysis, may interfere with niacin metabolism. 

Niacin deficiency is not commonly reported in DKD, with no cases of pellagra having been reported in DKD patients previously. Little is currently known regarding the pathophysiological impact of niacin deficiency in DKD and vice-versa. Considerations of a potential association between niacin and its action to restrict intestinal phosphorus absorption were noted though—previous randomized studies describing reduced serum phosphate levels with niacin supplementation amongst kidney failure patients (including those with DKD) receiving maintenance hemodialysis (HD) [69,70]. No difference was observed regarding serum parathyroid hormone (PTH), fibroblast growth factor-23 (FGF-23), calcium, or vitamin D between the niacin supplementation and control groups [69,70]. More research is needed to determine the relationship between niacin and DKD, and biochemical markers of DKD severity.

## 5. Vitamin B5 (Pantothenic Acid)

Vitamin B5 (pantothenic acid), otherwise known as “chick anti-dermatitis factor”, stems from the combination of pantoic acid and beta-alanine [71]. Pantothenic acid is present in large amounts across a wide range of foods—cod ovaries, egg yolk, fresh vegetables, liver, kidney, royal bee jelly, and tuna [72]. The formation of CoA and acyl-carrier proteins (ACPs), which carry and transfer acetyl and acyl groups, stems from pantothenic acid [71]. Several biochemical steps are involved in the conversion from pantothenic acid to CoA. Pantothenic acid, adenosine monophosphate, and beta-mercaptoethylamine are present in CoA [71]. The synthesis of ACP and many other compounds, including amino acids, cholesterol, fatty acids, gamma-aminolevulinic acid, various neurotransmitters, and steroid hormones [71]. CoA is also required for energy extraction during the oxidation of amino acids and beta-oxidation of fatty acids [71]. Being a precursor/component of CoA, pantothenic acid has a key role in the acetylation of histones, microtubules, proteins and in the acylation of proteins (the ACPs) with fatty acids, mainly myristic and palmitic acids [71,73]. The acetylation and acylation of proteins affect both their structure and activity. Following CoA hydrolysis, pantothenic acid is liberated and urinary excreted [71,73]. Pantothenic acid metabolism and actions can interact with several medications, namely tetracycline and cholinesterase inhibitors—the intestinal absorption and effectiveness of tetracyclines would be affected by pantothenic acid, and it may also accentuate the physiological effects of cholinesterase inhibitors [18].

Our present understanding of the pantothenic acid physiology and action in diabetes and DKD is vague and few studies in this respect have been conducted. No specific syndromes related to low pantothenic acid levels have been reported in diabetic and DKD patients. Preliminary findings from a rat study highlighted increased pantothenic acid levels following dexpanthenol administration in the early phase of diabetes, reiterating the potential significance of pantothenic acid in affecting DKD outcomes [74]. Some small observational studies involving DKD patients on kidney replacement therapy (maintenance HD patients and continuous ambulatory peritoneal dialysis (CAPD)) found pantothenic acid levels were higher alone without supplementation compared to normal controls, despite the original report involving six maintenance HD patients that suggested the reverse [75,76]. Findings from the PROGREDIR study, which investigated associations between dietary intake and coronary artery calcification in non-dialysis CKD, noted significant associations between increased serum pantothenic acid levels and increased coronary calcification scores amongst non-dialysis CKD/DKD patients [77]. In summary, pantothenic acid status in patients with advanced DKD and those receiving kidney replacement therapy remains controversial. Further basic research on the physiological relationships is required to address this debate.

## 6. Vitamin B6 (Pyridoxine) 

Three derivatives of the pyridoxine ring—pyridoxine, pyridoxal, and pyridoxamine forms vitamin B6 (pyridoxine). Phosphorylation in the 5′ position is a vital component of pyridoxine activity, in which pyridoxal-50-phosphate (PLP) and pyridoxamine-50-phosphate are its active coenzyme forms [78,79,80]. Pyridoxine source is mainly located within plant-based foods, such as avocado, banana, lentils, potatoes, soybean (in cooked form), walnuts, and wheat bran whilst in contrast, pyridoxal and pyridoxamine are mainly sourced from animal food products such as chicken breast (raw), ground beef, and tuna [80,81]. Pyridoxine, pyridoxal, and pyridoxamine are absorbed in the jejunum through a non-saturable, passive process and phosphorylated in the liver by pyridoxine kinase, in which this process requires zinc and ATP [78,79,80]. Also occurring within the liver following phosphorylation—alkaline phosphatase dephosphorylates pyridoxine (those in phosphorylated forms), which leads to pyridoxal and then by the irreversible action of a FAD-dependent aldehyde oxidase, to 4-pyridoxic acid (4-PA) [78,79,80]. The biochemical actions of flavin mononucleotide oxidase allows PLP to be generated from the other two vitamers, and PLP Is transported in plasma bound to albumin and in red cells bound to hemoglobin [78,79,80]. Majority of PLP would be stored in skeletal muscle bound to glycogen phosphorylase [78,79,80]. PLP is the coenzyme to more than 140 enzymatic reactions in the body [78,79,80,81,82,83,84]. It is particularly involved with enzymes relating to amino acid and lipid metabolism [83,84,85]. A Schiff base is formed by PLP with the epsilon-amino group of lysine in many enzymes [86]. The Schiff base alters the charge on the rest of the PLP molecule and strongly increases its reactivity, particularly towards other amino acids [86]. Through its role of facilitating transamination and glycogen phosphorylation amongst other mechanisms, pyridoxine is essential for the process of gluconeogenesis during niacin formation via the PLP-dependent kynureninase, which transforms tryptophan to niacin [87]. Another important purpose is to achieve normal erythrocyte metabolism as much as possible, by acting as coenzyme for transaminase and influencing the hemoglobin affinity for oxygen [87]. Pyridoxine also plays a significant role in facilitating the synthesis of several neurotransmitters, as well as modulating the actions of certain hormones through the binding of PLP to steroid receptors [88,89]. 

Several inherent factors, without considering the effect of diabetes and DKD at this stage, may influence PLP levels. Older people are more likely to have lower PLP levels compared to younger people, whilst women would usually have lower PLP levels compared to men [90]. This is most likely explained by the differences in muscle mass with age and gender. An inverse correlation is observed between serum PLP levels and dietary protein intake [90,91,92]. Smoking reduces overall pyridoxine levels [92]. Numerous medications may interfere with pyridoxine action and metabolism—cycloserine, ethionamide, isoniazid, penicillamine, and theophylline blocks the synthesis of pyridoxine-L-phosphate; pyridoxine could reduce phenytoin metabolism and thereby increase the risk of seizures; pyridoxine can also decrease the effectiveness of levodopa and increase parkinsonian symptoms [93]. 

Observational studies have noted pyridoxine deficiency in patients with T2DM and DKD. Nix and colleagues [94], investigating pyridoxine status in adults with and without incipient nephropathy secondary to T2DM, in addition to thiamine and cobalamin and its related vitamers and biomarkers (including total homocysteine, methylmalonic acid), have found reduced PLP levels alongside pyridoxine deficiency amongst 120 adults with T2DM (including 46 patients with microalbuminuria) in comparison to non-diabetic controls. The authors concluded that incipient nephropathy was associated with more pronounced alterations in pyridoxine metabolism and progression of endothelial dysfunction and inflammation as a result of pyridoxine deficiency. A cause–effect relationship between diabetes, DKD, and pyridoxine has been extensively investigated. The role of inflammation in the pathogenesis of diabetes was shown to be an important cause of decline in PLP levels [80,95,96,97]. It is proposed that in diabetes, decline in serum PLP levels may be attributed to a combination of factors—from increased PLP mobilization to the site of inflammation, increased demand by the PLP-dependent enzymes involved in the tryptophan kynurenine pathway due to diabetic effects on tryptophan metabolism, to immune cell proliferation [97,98,99]. This may develop into a vicious cycle where pyridoxine deficiency impairs insulin and glucagon secretion mechanisms, as well as lipid metabolism due to effects on the homocysteine pathway resulting in hyperhomocysteinemia (PLP is a cofactor for cystathionine-synthase and cystathionine-lyase, which are involved in the metabolism of this compound), and increasing oxidative stress [80,85,98,100,101]. The inverse associations between pyridoxine deficiency in diabetes and oxidative stress is of immense pathophysiological significance, as it predisposes to increased advanced glycation end-product (AGE) formation causing microvascular damage, leading to DKD, diabetic neuropathy, and retinopathy, as well as DNA damage and neoplasia risks from elevated reactive oxygen species (ROS) formation [102,103]. Specific to those who have developed DKD, another pathophysiological abnormality that has been speculated with pyridoxine deficiency involves the altercation to immune function and homeostasis [104]. Reduced numbers of blood granulocytes and lymphocytes, decreased lymphocyte maturation, reduced blastogenic response of lymphocytes to mitogenic stimuli, delayed cutaneous hypersensitivity, and decreased antibody production have been observed [105,106,107]. Furthermore, pyridoxine deficiency may contribute increased serum oxalate levels due to increased formation of oxalate, with pyridoxine being a coenzyme for the transamination of glyoxylate to glycine [108]. Oxalate synthesis is hugely contributed by ascorbic acid, and pyridoxine deficiency also has a key role in this process [109]. Oxalate is a uremic toxin that is urinary excreted normally, and very little oxalate is degraded in vivo in humans [110]. The combination of DKD and pyridoxine deficiency has considerable physiological significance in elevating serum oxalate levels [106,108,109,111].

## 7. Vitamin B8 (Biotin)

Vitamin B8 (biotin) is a bicyclic compound containing an ureido and tetrahydrothiophene ring, which could be synthesized by intestinal flora, though it is believed this alone will not be enough to meet the daily demands of biotin in humans. High biotin content food sources include egg yolk, liver, soybean, and yeast. Other foods which contain moderate amounts of biotin include cereals, legumes, and nuts. Biotin absorption mainly occurs in the jejunum [112,113]. This process requires ingested proteins releasing biocytin, and also the separation of lysine and biotin, which are each absorbed by a diffusive saturable process, through the action of biotinidase [112,113]. The main role of biotin in the human body is as a carbon dioxide carrier, as biotin is the coenzyme for 5-carboxylases (i.e., acetyl-CoA carboxylase, pyruvate carboxylase, propionyl-CoA carboxylase, and B methylcrotonyl-CoA carboxylase) where it also covalently binds to the epsilon-amino group of a lysine residue of carboxylases [112,113,114]. Hence, it plays an important role in amino acid, carbohydrates, and fatty acid metabolism [114]. There are numerous medications that may interrupt biotin absorption, its metabolism, and other actions. Antibiotics may reduce the intestinal microflora responsible for biotin production, and hence reduce biotin levels [18]. Anticonvulsant medications such as carbamazepine and primidone, if taken long-term, may reduce biotin levels through its interference with biotin absorption at the intestinal brush border [115]. Biotin absorption at the intestine may be impaired by avidin, a glycoprotein that strongly binds to biotin. Free biotin and its metabolites are excreted in the urine [116].

The physiological associations between biotin and diabetes have been well-investigated. Numerous in vitro and in vivo studies have demonstrated that biotin could stimulate pancreatic islet glucokinase activity and expression, increase insulin secretion, and induce insulin receptor synthesis in non-diabetic states, whilst considering the significance of biotin as an efficient ameliorator of hyperglycemic status in diabetes [117]. Although the mechanism of hyperglycemia is different between T1DM and T2DM, biotin plays an important role in glucose homeostasis for both types of diabetes. An in vivo study investigating the effects of biotin in rodent pancreatic islets confirmed biotin augments the function and proportion of beta-cells whilst also suppressing mRNA expression of neural cell adhesion molecule-1 (NCAM-1) (NCAM-1 is an adhesion protein participating in the conservation of islet architecture) [118]. These actions prevent the onset of diabetes and in biotin deficiency, there is reduced glucose tolerance with the malfunctions of this mechanism in maintaining glucose homeostasis. Considering the key role of biotin regulating the glucokinase gene at the transcriptional stage in starved states, energy production could be impaired by biotin deficiency, in which there is decreased glucose utilization and oxidative phosphorylation [118]. Insulin secretion and expression were found to be hindered with biotin deficiency, whilst this increased again with biotin supplementation [118]. High levels of biotin administration may compensate for subnormal insulin exposure by suppressing FOXO1 levels [119,120]. 

Biotin may counteract the effects of uremic toxins on tubulin (it has been shown to impair tubulin polymerization, which leads to cellular microtubule formation under normal conditions) [121]. It is expected that biotin deficiency would be correlated with CKD/DKD severity considering this. However, several clinical observational studies in the past have displayed findings which suggested the reverse, bringing controversy to this logic [122,123]. Comparatively few studies have evaluated the physiological role of biotin on CKD/DKD progression, and further study is needed. Aldahmash and colleagues [124] investigated biotin and its protective role on oxidative stress and kidney pathological changes in T1DM mice. Male Swiss albino mice were randomly divided into three groups—the control group received saline whilst T1DM was induced in the second and third groups by intraperitoneal injection of streptozotocin as a single dose (150 mg/kg). The second group remained as an untreated diabetic group, and the third group received 15 mg/kg daily oral dose of biotin for 12 successive days. In the second and third groups, biochemical results showed significant elevation in blood glucose and urea levels compared to the control groups. There is an increase in glomerular areas and a decrease in glomerular cellularity in the second and third groups. Histopathological results showed severe alterations in the second group, represented by distorted glomeruli, inflammatory cells, and giant macrophages. In addition, there was an intense immune-reaction response toward acrolein indicator of oxidative damage. Upon biotin administration, the above-mentioned histopathological changes were reduced, and there was also diminishing in the acrolein reactions of oxidative damage. The authors thus noted with these findings potential significant associations between biotin and oxidative stress (as induced by streptozotocin in this instance) in kidneys, and DKD progression. 

## 8. Vitamin B9 (Folate) 

Vitamin B9 (folate), a family of structurally related compounds that have pteroylglutamic acid at its core, has major metabolic functions in the acceptance, redox processing, and transfer of one carbon (includes methyl, formyl, methylene, and methenyl groups) [125,126]. Folic acid (which is also known as pteroylmonoglutamic acid) is not usually found in natural form or food sources [125,126]. Due to its chemical stability and low cost of production, folic acid is commonly synthesized, used as a vitamin supplement and fortificant in food products [127]. A pteridine moiety, para-aminobenzoic acid, and glutamic acid form the three subunits of commercially synthesized folic acid [126]. There are many foods which contain folate—fruits, green vegetables, meats, liver, and yeast amongst them [125,126]. Most naturally occurring food folates are pteroylpolyglutamates that contain from one to six additional glutamates and are joined to the gamma-carboxyl of glutamate [125,126]. Tetrahydrofolate (THF) is a reduced form of folic acid that is present both in foods and in the human body [125,126]. Folates are very sensitive to oxidation, and food sources of folate could be destroyed by food processing such as canning and refining, and extensive cooking [128]. Folate absorption typically occurs at the proximal first third of the small intestine. Conjugase action is important to transform folate polyglutamates to folate monoglutamates (5-methyl-THF, formyl-THF, or dihydrofolates). Conjugases are present in the brush border of enterocytes [125,126,129]. The glutamate carboxypeptidase II is another enzyme at the intestinal brush border that is involved in folate polyglutamate metabolism [128]. An H475Y DNA variant coding for this enzyme has been identified from previous studies, where a reduction of 53% of enzyme activity was found to associate with low folate levels and hyperhomocysteinemia [128,130]. Folate membrane receptors, carriers, and exit pumps in cellular membranes play an important role in the cellular transport of folate [125,126]. Most folates are stored in the body as folate polyglutamates. Conjugase action is required to synthesize polyglutamates into monoglutamates, its biologically active monoglutamate form [125,126]. Ultimately, folate polyglutamates may yield physiological actions themselves. The majority of serum folate is free or loosely bound to non-specific carriers [125,126]. A specific cell membrane receptor protein is required to deliver folate from serum to tissues. Vitamin B12 (cobalamin), discussed in the next section, is involved in transmethylation reactions such as folate and a vital component in the transport and storage of folate. Excretion of free folate and metabolites of folic acid occurs in urine and bile [125,126]. Alcohol consumption impairs the folate enterohepatic cycle, which helps to preserve the body’s pool of folates [131]. 

**Figure 2:**
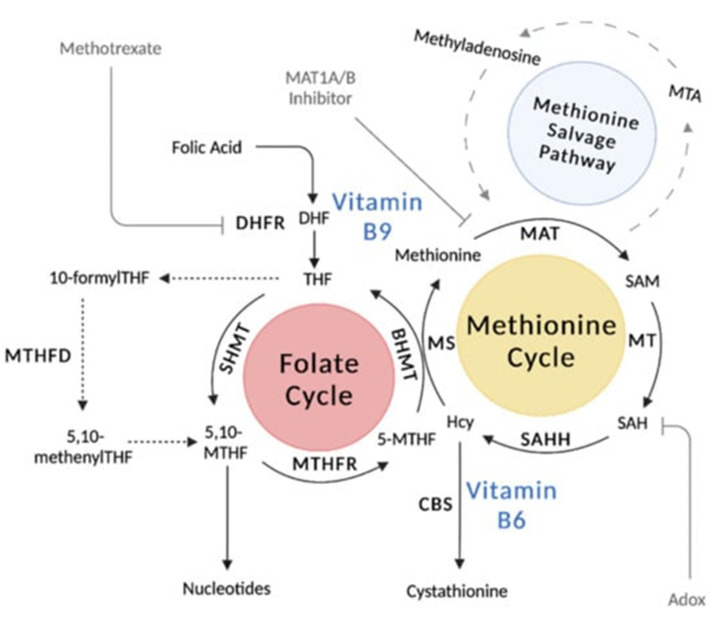
Processes and pathways of one-carbon metabolism. Figure obtained from [132].

The physiological action of one-carbon unit transfers basically summarizes the fundamental role of folate (Figure 2) [132]. Folic acid is needed for DNA synthesis. 5,10-methylene THF requires the vitamin B12 (cobalamin)-dependent transmethylation of homocysteine methionine, delivers its methyl group to deoxyuridylate, and is subsequently transformed to thymidylate [133]. Megablastosis occurs when there are defects in DNA synthesis—this is evident in all human replicating cells but is most apparent amongst cells in the bone marrow [134]. Folate plays a significant role in the metabolism of amino acids, in particular for amino acids, which are methyl donors or receivers [125,126,135]. This includes the interconversion of glycine and serine, the transformation of homocysteine to methionine, and the conversion of histidine to glutamic acid. Another role of folate is for purine synthesis in tRNA methylation [136]. 

Various medications inhibit the actions of folate and conjugases. Methotrexate is a noticeable one, this being a medication with anti-folate action that would likely cause folate deficiency in toxic levels [137]. Antacids, bile acid sequestrants, carbamazepine, histamine-2 receptor antagonists, non-steroidal anti-inflammatory medications, proton pump inhibitors, sulfasalazine, and triamterene decrease folate intestinal absorption. Another key factor affecting folate metabolism is MTHFR gene polymorphisms, in which the main variants are at positions 677 (MTHFR 677C.T), 1298 (MTHFR 1298A.C), 1317 (MTHFR 1317T.C), and 1793 (MTHFR 1793G.A) [138]. Although numerous studies over the years have demonstrated associations between MTHFR gene polymorphisms and folate deficiency, the exact mechanisms are not clear and require further investigation [139,140]. 

Mice studies comparing chronic folate deficiency-diet-fed mice and controls have demonstrated the impact of folate deficiency on glucose intolerance and insulin resistance [141]. It has been found that folate deficiency significantly decreased serum insulin levels [141]. The pathophysiological process is explained by another in vitro study, noting that folate deficiency triggers oxidative-nitrosative stress, and subsequently endoplasmic reticulum stress in the insulin-producing pancreatic islet RINm5F cells, resulting in RINm5F cell apoptosis as well as impairment of the biosynthesis and secretion of insulin [142]. Furthermore, the effect of folate deficiency on disorders of the homocysteine metabolic pathway, resulting in elevated homocysteine levels, is also a source of increased insulin resistance [143]. Elevated serum homocysteine levels lead to increased levels of its metabolite homocysteine thiolactone, which inhibits insulin receptor tyrosine kinase activity, decreases phosphatidylinositol 3-kinase activity, and attenuates the phosphorylation of Akt [143,144]. These mechanisms explain how folate deficiency can cause insulin resistance and diabetes. 

Currently, there are no established direct links between folate deficiency and DKD, despite more evidence suggestive of causative associations between folate deficiency and diabetic retinopathy. Effects of folate deficiency on DKD progression could be caused indirectly from hyperhomocysteinemia [133]. Hyperhomocysteinemia induces inflammatory and oxidative stress pathways leading to endothelial dysfunction and progressive changes in the vasculature, eventually resulting in microvascular damage within the glomerulus [145,146]. Hyperhomocysteinemia can cause glomerular cell sclerosis and induce acute kidney injury through plasma and tissue adenosine level reduction [147,148]. As a result, there would be greater levels of vascular smooth muscle cell proliferation, vascular sclerosis, and mesangial expansion within the glomeruli, which will exacerbate podocyte dysfunction and enhance the progression of kidney fibrosis [147,148]. Another interesting finding of note is that amongst DKD patients, patients with an MTHFR 677 C>T polymorphism C677 mutation and TT genotype were found to be greatly susceptible to folate and cobalamin deficiency, though the mechanisms of this observation are not clear at present [149]. It was found that the coexistence of homozygosity for the C677T mutation or TT genotype alongside folate and cobalamin deficiency significantly increased risks of endothelial dysfunction and cardiovascular risks [149].

## 9. Vitamin B12 (Cobalamin) 

Vitamin B12 (cobalamin), also known as the anti-pernicious anemia factor, is the extrinsic factor sourced from dietary intake and combined with the intrinsic factor present in gastric juice for intestinal tract absorption [150,151]. Cobalamin is formed by a corrin nucleus, a nucleotide, and a cobalt atom [152]. Cobalamins are unstable in light and could be destroyed by strong oxidation and reduction agents [150,151]. A pharmaceutical form of cobalamin, cyanocobalamin, has been isolated from liver extracts. Metabolically active forms of cobalamin include coenzyme B12 and methylcobalamin [150,151]. All forms of cobalamin in large doses are equally effective [153]. Bacteria-synthesized cobalamin remains the only natural source of cobalamin. Animal tissues could be abundant with cobalamins—mostly liver, meat, and seafood, with lesser amounts in egg yolk and milk [154]. Few sources of cobalamin are present in fruit and vegetables [155]. The process of cobalamin absorption begins with the combination of free cobalamin with salivary peptide binder, with this complex released into the small intestine by trypsin where it combines with the intrinsic factor [156,157]. Binding of the cobalamin-intrinsic factor complex to a receptor on the brush border of ileum mucosal cells allows for its absorption to occur [156,157]. If cobalamin is administered as large pharmacological doses, it may be absorbed via passive diffusion by the small intestine [157]. There are three binding proteins, transcobalamins I, II, and III, which participate in cobalamin transport in plasma and in the in vivo storage of cobalamin [158]. Cobalamin stores can typically constitute up to several milligrams, and this amount may prevent cobalamin deficiency for several years if intestinal absorption of cobalamin is halted [122]. Cobalamin is not catabolized during its metabolic cycle and is biliary excreted with an efficient enterohepatic cycle [131]. As briefly noted in the previous sub-section, cobalamin plays an instrumental role in folate metabolism and in methyltetrahydrofolate demethylation and homocysteine methylation [125,126,131,133]. This is a key step in the pathway for the THF regeneration, which allows for DNA synthesis by thymidylate synthesis and for folate delivery to body tissues [125,126,131,133]. Therefore, cobalamin deficiency can lead to the absence of demethylation of methyltetrahydrofolate, resulting in folate deficiency subsequently as well. 

There are numerous medications that can interfere with cobalamin metabolism and its actions. Anticonvulsants, bile acid sequestrants, chemotherapy medications, colchicine, histamine-2 antagonists, metformin, and proton pump inhibitors may reduce cobalamin levels at various points during its metabolic pathway [159]. In addition to the potential of MTHFR gene polymorphisms affecting cobalamin (and folate) levels, inborn genetic errors that lead to cobalamin deficiency have also been described. Cobalamin C deficiency could be caused by methylmalonic aciduria cblC type with homocystinuria (MMACHC) gene mutations [160,161]. These mutations lead to the deficit of two active coenzyme derivatives of cobalamin, adenosylcobalamin, and methylcobalamin [160,161]. As a result, cobalamin C deficiency may lead to some cases of atypical hemolytic uremic syndrome (HUS) in children and young adults [162]. Histological evaluation of these cases found the glomerular basement membrane with vacuolated appearances and IgM staining of the glomerular capillary wall, in addition to the classic HUS-related microangiopathic lesions [161]. Clinical improvement is observed following correction of cobalamin C deficiencies with parenteral injections of hydroxocobalamin [161]. 

Cobalamin deficiency in diabetes has been highlighted in observational studies, more frequently so over the past decade. However, the purported mechanisms behind why cobalamin deficiency occurs in diabetes, particularly in T2DM, are more as a consequence of drug effects rather than diabetes itself [163,164]. Metformin-induced cobalamin deficiency among patients with T2DM may be explained by alterations in small bowel motility, which stimulates bacterial overgrowth and consequential cobalamin deficiency [165,166]. Deficiency may also occur in this instance due to competitive inhibition or inactivation of cobalamin absorption, alterations in intrinsic factor levels, and interaction of metformin with the cubulin endocytic receptor [165,166,167,168]. Furthermore, metformin has also been shown to inhibit the calcium-dependent absorption of the cobalamin-intrinsic factor complex at the terminal ileum, which can be reversed with calcium supplementation [169,170]. In contrast to T2DM, T1DM is an autoimmune condition that results from destruction of insulin-secreting pancreatic beta cells. It is associated with other organ- and non-organ-associated autoimmune and endocrine conditions, namely chronic autoimmune gastritis and pernicious anemia. Amongst patients with T1DM, the prevalence of chronic autoimmune gastritis and pernicious anemia increases up to five-fold [171]. Cobalamin deficiency as a result of pernicious anemia occurs frequently in patients with T1DM—patients with T1DM actively exhibit autoantibodies to intrinsic factor type 1 and 2 and parietal cell antibodies, in particular those with glutamate decarboxylase-65 antibodies and HLA-DQA1*0501-B1*0301 haplotype [172]. 

Similarly to folate, there is a lack of evidence that demonstrates a direct relationship between cobalamin deficiency and CKD/DKD progression outside of the hyperhomocysteinemia pathway (the mechanistic effects of hyperhomocysteinemia upon CKD/DKD progression is covered in the previous sub-section). It was previously suggested that the chronic inflammatory state of CKD/DKD will have reduced transcobalamin II production due to reduced cobalamin intake from the circulation to peripheral tissues. In response, there would be increased synthesis of transcobalamin I and III, which elevates the plasma cobalamin levels [173,174]. This proposed feedback mechanism requires further validation. Furthermore, direct effects of cobalamin deficiency upon oxidative stress build-up outside of the hyperhomocysteinemia pathway has also been considered in diabetic and CKD/DKD states, albeit with controversial conclusions as to whether cobalamin deficiency is related to increased pro-oxidant and decreased antioxidant status [175,176,177]. There remains a paucity of prospective studies in this aspect, and further research is warranted. 

## 10. Summary and Future Directions 

Our knowledge of the biochemical and physiological properties of vitamin B and the physiological associations between vitamin B deficiency and DKD has certainly increased over recent decades. Table 1 summarizes the evidence presented in this review article in relation to the associations between each vitamin B sub-form and DKD. There remain sizeable limitations in our knowledge base within this topic, more significantly so for some vitamin B sub-forms than others. The importance of pursuing a greater understanding on the physiological relationships between vitamin B, diabetes and kidney disease is understated, with continuing discoveries at a basic level. Going forward, further research is needed to address knowledge gaps in relation to the properties of vitamin B and their associations with DKD in vitamin B-deficient states. Advancements in our basic scientific understanding may lead to a greater abundance of high-level evidence in guiding and optimizing a translational micronutrition care approach for the DKD patient population.

## Figures and Tables

**Table 1:** Summary of evidence describing physiological associations between each vitamin B sub-forms and diabetic kidney disease.

Vitamin B Sub-Form	Summary of Evidence
Thiamine (Vitamin B1)	Epithelial cells in the kidney tubules are likely to have decreased expression of thiamine transport protein, isoform 1 (THTR1) in a hyperglycemic environment, stimulating greater levels of renal thiamine clearance (Mazzeo et al., Larkin et al., Bukhari et al.) [33,34,35]. Both rat and human plasma samples identified decreased levels of thiamine concentration in subjects with diabetes (Rabbani et al., Babaei-Jadidi et al., Patrini et al.) [31,36,37,38] Thiamine deficiency can cause increased accumulation of soluble vascular cell adhesion molecule-1 and von Willebrand factor, in which inflammatory cytokines and growth factors are a key part, contributing towards the loss of glomerular endothelial glycocalyx in diabetic kidney disease (DKD) and severe persistent microalbuminuria (Thornalley et al., Rabbani et al.) [29,41]. Abnormal expression of transketolase with thiamine deficiency weakens enzymatic defense against glycation and its ability to counter metabolic stress, resulting in further acceleration of vascular dysfunction and progression of DKD (Hammes et al., Karchalias et al., Xue et al., Brownlee et al.) [43,44,45,46].
Riboflavin (Vitamin B2)	Observations from mice studies noted associations between riboflavin, oxidative stress status, and deoxyribonucleic acid (DNA) damage (Jankowska et al., Kim et al., Alam et al.) [51,52,53]. Riboflavin improved urea, creatinine, and antioxidant status in the kidneys (Alam et al.) [53]. There is a greater recovery of glutathione reductase, as well as reductions in lipid peroxidation, protein oxidation, GST, and sulfhydryl levels in the kidneys following riboflavin supplementation (Aguilar et al., Can et al.) [54,55]. Mice study highlighted an improved histopathological outlook in the kidneys following riboflavin supplementation, in which mice with more severe diabetes exhibited less diabetes-induced structural aberrations attributed to the effects of riboflavin. Riboflavin dose-dependent cellular DNA damage recovery is also found from histopathological analysis (Alam et al.) [53].
Niacin (Vitamin B3)	Niacin supplementation restricts intestinal phosphorus absorption and may facilitate towards lowering serum phosphate levels in chronic kidney disease (CKD)/DKD (Lenglet et al., Zhang et al.) [69,70].
Pantothenic Acid (Vitamin B5)	Conclusions in relation to pantothenic acid status in patients with DKD and those receiving kidney replacement therapy and the pathophysiological associations between pantothenic acid and DKD remains controversial. Most of the studies conducted are small in number of samples/patients. Further basic research is required (Tutun et al., Poyan Mehr et al., Mackenzie et al., Machado et al.) [74,75,76,77].
Pyridoxine (Vitamin B6)	Decline in pyridoxal-50-phosphate (PLP) levels from pyridoxine deficiency may affect various metabolic pathways leading to increased inflammatory responses and oxidative stress. A reduced supply of PLP-dependent enzymes may affect tryptophan metabolism and dysregulate insulin and glucagon homeostasis, as well as lipid metabolism and the homocysteine pathway (Stach et al., Endo et al., Shen et al., Friso et al., Mascolo et al., Oxenkrug et al., Fields et al., Hsu et al., Pusceddu et al.) [80,85,95,96,97,98,99,100,101]. These effects may lead to increased advanced glycation end-product (AGE) formation causing microvascular damage as observed in DKD and other intrinsic kidney diseases, as well as DNA damage and neoplasia from elevated reactive oxygen species (ROS) formation (Wotherspoon et al., Chen et al.) [102,103]. Pyridoxine deficiency may result in alterations to immune function and homeostasis specific to subjects with DKD (Mikkelsen et al.) [104]. Pyridoxine deficiency alongside the pathophysiological processes of DKD may contribute towards increased oxalate formation and subsequently increased serum oxalate levels (Mydlik et al., Morgan et al.) [108,109].
Biotin (Vitamin B8)	There remains inconclusive debate as to the effects of biotin action upon uremic toxins on tubulin (Jung et al., Descombes et al., Fujiwara et al.) [121,122,123]. Study involving type 1 diabetes mellitus mice observed the protective role of biotin in reducing oxidative stress and kidney histopathological progression (Aldahmash et al.) [124].
Folic Acid (Vitamin B9)	Direct links between folate deficiency and DKD are currently unestablished. Effects of folate deficiency on DKD progression is thought to be mainly caused indirectly from hyperhomocysteinemia, where hyperhomocysteinemia induces inflammatory and oxidative stress pathways leading to endothelial dysfunction and progressive changes in the vasculature, eventually resulting in microvascular damage within the glomerulus (Wu et al., Jiang et al., Li et al.) [133,145,146]. Hyperhomocysteinemia can also cause sclerosis in glomerular cells and induce acute kidney injury through reducing plasma and tissue adenosine levels, in which vascular smooth muscle cell proliferation, advances vascular sclerosis and mesangial expansion within the glomeruli which may eventually exacerbate podocyte dysfunction and kidney fibrosis (Pecoits-Filho et al., Garcia-Fernandez et al.) [147,148]. Genetic polymorphisms (i.e., MTHFR 677 C>T polymorphism C677 mutation and TT genotype) alongside folate deficiency is found to significantly increase risks of endothelial dysfunction and cardiovascular events, though the mechanisms of this trend require further elucidation (Achour et al.) [149].
Cobalamin (Vitamin B12)	Proposed physiological associations between the role and actions of transcobalamin (I, II, and III) and chronic inflammation resulting in DKD progression require further validation (Seetharam et al., Park et al.) [173,174]. Direct effects of cobalamin deficiency on oxidative stress has also been considered in diabetes and CKD/DKD, but with controversial conclusions as to whether cobalamin deficiency is related to increased pro-oxidant and decreased antioxidant status. Further work is needed (Obeid et al., Nowotny et al., van de Lagemaat et al.) [175,176,177].

## Data Availability

No new data were created for this article.
